# Combination of long-acting HIV fusion inhibitor albuvirtide and LPV/r showed potent efficacy in HIV-1 patients

**DOI:** 10.1186/s12981-016-0091-1

**Published:** 2016-02-10

**Authors:** Hongwei Zhang, Ronghua Jin, Cheng Yao, Tong Zhang, Meixia Wang, Wei Xia, Haiyan Peng, Xiaojuan Wang, Rongjian Lu, Changjin Wang, Dong Xie, Hao Wu

**Affiliations:** Beijing You’an Hospital, Capital Medical University, 100069 Beijing, People’s Republic of China; Nanjing Frontier Biotechnologies Co. Ltd., 5 Qiande Road, Jiangning District, 211122 Nanjing, People’s Republic of China; Beijing Co-CRO Medical Development Co. Ltd., Beijing, People’s Republic of China; Center for Infectious Diseases, Beijing You’an Hospital, Capital Medical University, 8 Xitoutiao, Youanmenwai St, Fengtai Dist, 100069 Beijing, People’s Republic of China

## Abstract

**Background:**

Long acting antiretroviral drugs represent a promising approach for chronic treatment of HIV infection. Here, we study the efficacy and safety of albuvirtide (ABT), an HIV-1 fusion inhibitor with a half life of 11–12 days in human.

**Methods:**

ABT was evaluated in a 7-week, open-label and randomized trial, combining with LPV/r. Twenty HIV-1-infected adults were assigned to two dose groups, receiving ABT (160 or 320 mg) given weekly and LPV/r given twice daily.

**Results:**

At week 7, the decline of HIV-1 RNA from baseline was 1.9 (1.3–2.3) log_10_ and 2.2 (1.6–2.7) log_10_ copies/ml, and suppression of HIV-1 RNA to below 50 copies/ml was achieved in 11.1 % (1/9) and 55.6 % (5/9) patients, for the 160 and 320 mg dose group respectively.

**Conclusion:**

A clear dose-efficacy correlation of ABT was demonstrated. ABT combining with LPV/r is a promising two-drug regimen to be tested in larger patient population.

## Background

Current antiretroviral therapy (ART) requires strict life-long adherence to daily drug taking. Long-acting ART agents, capable of being administered on a weekly or less frequent basis, have the potential to improve adherence to therapy and allow a more forgiving time window of drug administration [1].

Albuvirtide (ABT) is a chemically modified peptide derived from the N-terminal sequence of HIV-1 gp41, and contains a 3-maleimimidopropionic acid (MPA) group in its 13th lysine side chain [2]. Previous studies demonstrated that upon intravenous injection ABT could rapidly conjugate with serum albumin and dramatically extend the peptide in vivo half-life from 1.7 to 25.8 h in rats and from 10.9 to 102.4 h in monkeys [2]. A recent study using pseudoviruses showed potent inhibitory activity of ABT against a broad spectrum of HIV-1 strains, including those commonly observed in China and some variants resistant to T20 [3]. A phase 1 single agent study in HIV-1 infected patients showed excellent safety profile of single and multiple dose of ABT, a half-life of 11–12 days, and a clear dose related antiviral activity [4].

In this study in naive HIV-1 patients, a novel two-drug regimen combining ABT and lopinavir/ritonavir (LPV/r) was tested, their drug–drug interaction investigated, and short-term safety and efficacy profiles characterized.

## Methods

### Study population

Antiretroviral treatment-naïve HIV-1-infected patients aged 18–50 years were eligible for this study if they had HIV RNA levels between 5000 and 1,000,000 copies/ml, CD4 cell counts more than 350 cells/μl, body weight more than 40 kg and body mass index (BMI) between 18.0 and 27.0 kg/m [2]. They had a normal level of albumin and no severe liver and kidney damage, and had not received any antiretroviral therapy for HIV and HBV or stopped antiretroviral therapy for more than 6 months. Exclusion criteria included acute HIV infection, severe opportunistic infections and tumors, severe diseases of digestive tract, hematology, metabolism, psychology and heredity, previous treatment of other HIV fusion inhibitors, drug abuse, pregnancy, and breastfeeding. Female patients with child bearing potential and heterosexually active male patients were required to use effective contraception during the study. The study was approved by the institutional ethics committee of Beijing You’an Hospital, and all potential subjects provided written informed consent before undergoing procedures.

### Study design

The study was a single-site, open-label, and randomized parallel study conducted in the Center for Infectious Diseases, Beijing You’an Hospital, Capital Medical University. Twenty antiretroviral treatment-naïve HIV-1 infected patients were enrolled and randomized into two dose groups (10 in each group) to receive ABT by intravenous infusion at dose levels of 160 or 320 mg, and LPV/r (400/100 mg) twice daily. On Day 5–7, ABT was given daily for 3 days, then given weekly till Day 40; and LPV/r was given from Day 1 to 46. The subjects were followed for a 47-day observation period. After the study all participants were treated with triple combination antiviral therapy containing tenofovir, lamifudine and LPV/r.

The plasma HIV-1 RNA was measured using a branched-chain DNA method (bDNA, version 3.0, Bayer Healthcare LLC, Diagnostics Division, Tarrytown, NY) with a detection limit of 50 copies/ml. CD4 cell counts were measured using a FACS count system (FACS Calibur, Becton Dickinson, USA).

### Statistical analysis

This was a pilot study without power calculations or sample size estimation. Data were presented as the mean ± SD if not specified. The efficacy and safety analyses were performed for the intent to treat (ITT) population, defined as all subjects who were enrolled in the trial and received at least one dose of study medication. All statistical analyses were performed using SAS version 9.2.

## Results

Twenty antiretroviral treatment-naïve HIV-1 infected patients (Table 1) were enrolled and randomized into two dose groups (10 in each group) to receive ABT (160 or 320 mg) and LPV/r. There were no significant differences in age, body weight, HIV viral load and CD4 cell count at baseline between the two groups. Nineteen subjects completed the study with one withdrawal due to lost to follow-up. Two patients whose baseline HIV viral load did not meet the inclusion criteria and their viral load data were excluded from efficacy analysis. All 20 subjects were included in the pharmacokinetic and safety analysis.

**Table 1:** Baseline characteristics of subjects

	160 mg ABT + LPV/r	320 mg ABT + LPV/r
Randomized and treated	10	10
Excluded	1	1
SS for safety analysis	10	10
ITT for efficacy analysis	9	9
Sex (male/female)	8/2	7/3
Race (Han/other)	9/1	10/0
Age (years)	31.9 (18~48)	37.4 (24~47)
Body weight (kg)	67.8 (53~88)	65.4 (53~76)
BMI (kg/m^2^)	22.8 (19~27)	22.8 (18~26)
Baseline HIV-1 RNA (log_10_ copies/ml)	4.27 (3.53~4.70)	4.27 (3.32~5.14)
Baseline CD4 (cell/μl)	517.0 (350~774)	566.6 (350~1070)

Data are presented as n or mean (range)

**Fig. 1:**
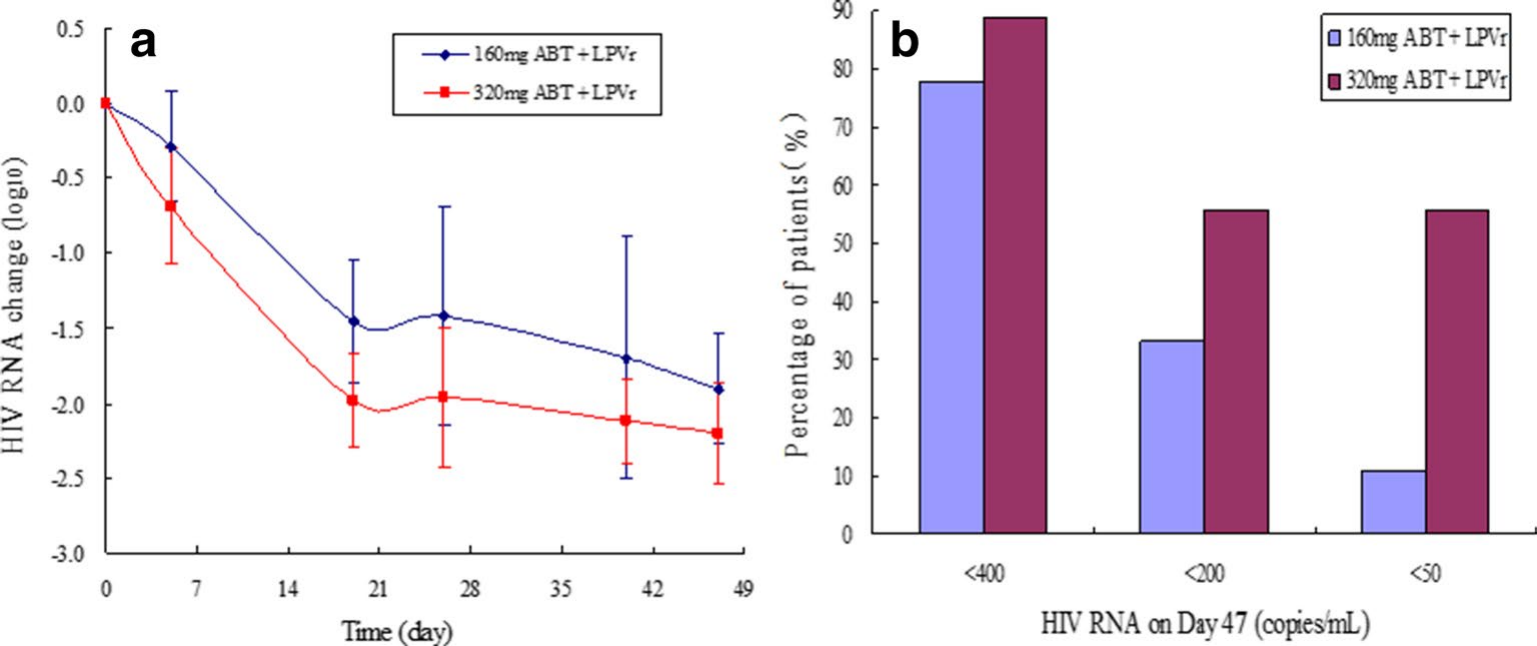
Anti-HIV activity of albuvirtide and LPV/r. **a** Mean change of plasma HIV RNA over time by dose group. **b** Percentage of patients with viral load <50, 200 and 400 copies/ml on Day 47

From Day 14 till the end of the study, the viral load (plasma HIV-1 RNA) of all patients decreased >1.0 log_10_ copies/ml. At the 47th day, the mean viral load decreased from baseline was 1.91 ± 0.36 log_10_ and 2.20 ± 0.33 log_10_ copies/ml for the 160 and 320 mg groups, respectively (Fig. 1a). The percentage of subjects with viral load <50 copies/ml was 11.1 % in the 160 mg group and 55.6 % in the 320 mg group (Fig. 1b). Pharmacokinetic/pharmacodynamic (PK/PD) relationship analysis showed that the decrease of HIV viral load was positively correlated with AUC_0-168h_, C_min_ and C_trough_ of ABT. After treatment with ABT and LPV/r for 47 days, the mean absolute CD4^+^ cell count change relative to baseline was –5 cells/μl for the 160 mg group and 52 cells/μl for the 320 mg group respectively.

The pharmacokinetic profile of co-administration of ABT and LPV/r showed weak or no interaction. The results will be reported elsewhere. There were no serious adverse events during the 47 days of treatment. Eight cases of adverse events were observed in 7 subjects in 160 mg group and nine cases in 8 subjects in 320 mg group, but only six cases were related to investigational regimen in each group. All adverse events were mild, mainly triglycerides level elevation, diarrhea, nausea and skin rash. No injection site reactions were found during the trial.

## Discussion

In chronic management of HIV infection with ART, one of the most challenges is poor patient adherence to treatment, which often results in treatment failure and emergence of drug resistance [5]. ABT is an experimental anti-HIV peptide targeting the HIV-1 envelop protein gp-41 at an area that’s different from that of T20. The 13th residue of ABT contains a MPA modification that allows ABT to react with serum albumin. This extends its half-life to 11–12 days in human while retaining anti-HIV activity [3]. Although long-acting AIDS drugs are much sought after, how to administer such molecules with current oral drugs remain to be studied.

In this phase 2 trial, we designed a two-drug regimen that includes a weekly given ABT and twice daily given LPV/r. It represents the first attempt to combine an approved, orally taken ART with a long-acting, injectable anti-HIV agent to treat HIV infected patients. For 47 days, the novel regimen was safe and exhibited potent anti-HIV activity. All treated patients, including one whose baseline viral load was >5 log_10_ copies/ml, showed >1 log_10_ copies/ml reduction of viral load. Importantly, data of the 320 mg group showed a trend of superior anti-HIV activity to that of the 160 mg group. This demonstrates clear contribution of ABT to the plasma HIV RNA reduction in the two-drug regimen.

ABT is the first long-acting antiretroviral drug developing in China. With the limitation of no long-acting drugs combined with ABT, we also chose LPV/r for the ART regimen in the phase III clinical trial, a 48-week, randomized, controlled, open-label, multicenter study to investigate the safety and efficacy of ABT, which may have potential as next-generation HIV fusion inhibitors targeting gp41 for clinical use [6].

## Conclusion

In summary, this 7-week study shows that ABT combined with LPV/r is safe and effective. Compared with regimens of 3–4 drugs, the two-drug regimen could offer a simplified therapy with better safety and less drug–drug interaction. The long half-life of ABT potentially allows a 3-day window for weekly administration and is more forgiving in adherence than daily taken drugs.
